# Case of Aneurismal Varix Treated Successfully during the Late War by Obliteration of the Vein

**Published:** 1868-09

**Authors:** N. Monmonier

**Affiliations:** Demonstrator of Anatomy Washington University, Balto., Md.


### ARTICLE V.

### *Case of Aneurismal Varix treated successfully during the late War by obliteration of the Vein.*

By Jno. N. Monmonier, M. D. Demonstrator of Anatomy Washington University, Balto., Md.

During the battle of July 3d, '63, at Gettysburg, Prvt. Aristide Mauvin, Co. K, 8th, La. Inf. C. S. A., whilst his command was charging Cemetery Heights, was wounded in the right upper extremity, the ball (Minie,) striking obliquely the Internal Condyle of the Humerus, split into several sharp and ragged fragments, one of which buried itself deeply in the upper portion or origin of the Flexor Carpi Ulnaris, Flexor Sublimis and Flexor Profundus Digitorum muscles.

After the extraction of the fragment, upon a careful examination I found the Internal Condyle considerably indented, without fracture and some little hemorrhage which occured *per saltem* and as I supposed, at the time, was caused by a wound either of the posterior Ulnar Recurrent or the Anastomotica Magna Arteries. Upon the application to the wound of a pledget of lint saturated with solution of Per Sulphate of Iron the hemorrhage soon ceased. This Soldier remained with his command and was not sent tothe General Hospital. About two weeks after the reception of the injury the wound had entirely healed and one morning he reported to me that there was a considerable tumor making its appearance near the point at which he had been wounded and which at times pulsated. After a careful manipulation I was satisfied that there existed an Aneurismal Varix (about the size of a pigeon egg) which was caused by the Brachial Artery and Median Basilic Vein having been either contused or slightly punctured by a fragment of the ball. The tumor was seated on the anterior surface of the extremity and to the inner side of the tendon of the Biceps muscle at the bend of the elbow.

Well knowing that the practical advantage derived, in a case of Varicose Veins aud Ulcers from the use of Acupressure and consequently obliteration of the distended veins, I determined to resort to this method of arresting the circulation in the vein. Accordingly the Median Basilic Veins was well isolated from the Brachial Artery and two steel pointed needles, about one inch and a half in length and about the diameter of an ordinary darning needle, were carefuly passed between the Artery and Vein, one above and the other below the tumor and twisted sutures of Surgeon's silk applied, in the form of figure of eight, around the head and point of each needle and the latter was armed with a small clamped buck-shot so as to prevent wounding of the integument. From day to day there was a perceptible diminution in the size as well as in the force of the pulsation of the tumor, and every alternate day the pressure upon the vein was increased by tightening the sutures. At the end of two weeks, after the introduction of the needles the pulsation had entirely ceased and there remained nothing of the tumor but an indurated cord. During the entire period in which the needles remained *in situ*, the patient suffered no inconvenience except occasionally there existed slight neuralgic pain, due as I suppose to some of the filaments of the Internal Cutaneous Nerve having been included between the needles and sutures. The man was returned to duty, perfectly relieved, and continued, as he had previously been, a good soldier and was subsequently killed in an engagement in Dec. '64, in front of Petersburg, Va.

